# Profile and satisfaction of oral pathology/medicine postgraduates: a national multicenter study

**DOI:** 10.4317/medoral.27436

**Published:** 2025-08-16

**Authors:** Luiz Miguel Ferreira, Marcos Paulo Maia-Lima, Samuel Trezena, João Pedro Santos Nascimento, Fabrício Emanuel Soares de Oliveira, Árlen Almeida Duarte, Daniella Reis Barbosa Martelli, Fábio de Abreu Alves, Roseana de Almeida Freitas, Jean Nunes dos Santos, Maria Cassia Ferreira de Aguiar, Marcio Ajudarte Lopes, Paulo Rogério Ferreti Bonan, Janete Dias Almeida, Hercílio Martelli-Júnior

**Affiliations:** 1 Department of Oral Diagnosis, School of Dentistry, University of Campinas, FOP-UNICAMP, Piracicaba, São Paulo, Brazil; 2 Postgraduate Program in Health Sciences/ Primary Health Care, State University of Montes Claros, UNIMONTES, Montes Claros, Minas Gerais, Brazil; 3 Head of Service, Oral Medicine Department, A. C. Camargo Cancer Center, São Paulo, Brazil; Stomatology Department, School of Dentistry, University of São Paulo, FO-USP, São Paulo, Brazil; 4 Postgraduate Program in Oral Science, Federal University of Rio Grande do Norte, UFRN, Natal, Rio Grande do Norte, Brazil; 5 Postgraduate Program in Dentistry and Health, School of Dentistry, Federal University of Bahia, UFBA, Salvador, Bahia, Brazil; 6 Department of Oral Pathology and Surgery, School of Dentistry, Federal University of Minas Gerais, UFMG, Belo Horizonte, Brazil; 7 School of Dentistry, Federal University of Paraíba, UFPB, João Pessoa, Paraíba, Brazil; 8 São Paulo State University (UNESP), Institute of Science and Technology, São José dos Campos Campus, São Paulo, Brazil; 9 Oral Pathology and Oral Medicine, School of Dentistry, State University of Montes Claros, UNIMONTES, Montes Claros, Minas Gerais, Brazil

## Abstract

**Background:**

This study aimed to evaluate the profile and professional satisfaction of postgraduate students in Oral Pathology (OP) and Oral Medicine (OM) enrolled in Stricto sensu programs across various Brazilian institutions.

**Material and Methods:**

A cross-sectional, multicenter study surveyed 139 students from seven universities using a digital questionnaire with 29 questions on sociodemographic, motivations, and satisfaction. Data collection occurred between August and October 2024.

**Results:**

Most participants were female (63.3%), aged between 25 and 30 years (59.7%), and enrolled in doctoral programs (55.4%). The most important reasons for pursuing postgraduate studies were knowledge enhancement (86.3%), personal satisfaction (84.9%), and financial factors (51.1%). Despite high satisfaction with their programs, and most of them (64%) feeling prepared for the job market, many students felt that job opportunities in OP/OM were limited. Older students reported greater satisfaction with the program (*p* = 0.020). Ethnicity influenced satisfaction with the pedagogical project (*p* < 0.001). Students with partners were more satisfied with the accessibility of the faculty (*p* = 0.004), as were those with children (*p* < 0.001). Job prospects were associated with satisfaction in several aspects, including the program (*p* < 0.001), the pedagogical project (*p* < 0.001) and the physical infrastructure (*p* = 0.034).

**Conclusions:**

Demographic factors and personal perspectives were associated with students' satisfaction with their postgraduate programs. Addressing employment opportunities and improving program infrastructures, pedagogical projects, and access to advisors/professors could further enhance students' satisfaction and career outcomes in OP/OM.

** Key words:**Dental education, oral pathology, oral medicine, career choice.

## Introduction

In Latin America, Oral Pathology (OP) and Oral Medicine (OM) are recognized fields with established academic programs. Master’s programs in these specialties are available in Argentina, Brazil, Mexico, Peru, Uruguay, and Venezuela, while PhD programs are limited to Argentina, Brazil, and Mexico (1,2).

In Brazil, OP and OM were formally recognized as dental specialties in 1971 and 1992, respectively (https://website.cfo.org.br/). The country plays a leading role, particularly in Latin America, in advancing these fields, with noTable postgraduate opportunities. Among Brazilian institutions, the Piracicaba Dental School at the University of Campinas (UNICAMP) is distinguished as the only public institution offering a Stricto Sensu postgraduate program exclusively dedicated to OP and OM. In addition, other institutions incorporate OP and OM as areas of concentration within broader research programs (https://sucupira.capes.gov.br/sucupira).

Despite its global significance, OM is often underrecognized by both professionals and patients, a trend also observed in Brazil, as reflected in the low number of specialists (https://website.cfo.org.br/). Research indicates that few dentists consider OP a viable career option, and OM is frequently not the first choice for dental students (3-5).

Between 2013 and 2021, 1220 Brazilian professionals completed their Stricto Sensu postgraduate programs in OP and OM, as reported by Bezerra *et al*. (2024) (6). This study highlights that most graduates pursued careers in teaching and research (44.3%). In clinical practice, the majority worked in private services (18.5%), followed by the public health system (10.6%), hospitals (6.8%), and military services (1.4%). However, the study did not address the profile and satisfaction of these professionals during their postgraduate study.

Understanding the profiles and satisfaction of students is important in the context of postgraduate education. An analysis of individual student profiles helps to gain in-depth insight into their experiences, obstacles and particular needs (7). Student satisfaction is also essential for the accreditation of educational institutions and maintenance of their positive image, as students are key stakeholders in the academic process (8). In some universities, annual research helps identify areas for improvement and guide future candidates. Nevertheless, academic satisfaction is also linked to better student outcomes, such as performance and motivation, while dissatisfaction can cause problems like burnout and anxiety (9).

Therefore, this study aims to assess the profile and level of professional satisfaction of Brazilian postgraduate students enrolled in Stricto sensu programs in OP and OM, as well as to identify the factors that influence professional choice in these areas.

## Material and Methods

- Design and Participants

This multicenter cross-sectional study was approved by the Research Ethics Committee (registration number 82618624.3.0000.5141) and adheres to the guidelines of the STROBE checklist for cross-sectional studies (10). The participants consisted of 188 postgraduate students in OP and OM enrolled in programs at various educational institutions in Brazil, including the University of Campinas (UNICAMP), Federal University of Minas Gerais (UFMG), Federal University of Rio Grande do Norte (UFRN), Federal University of Paraíba (UFPB), University of São Paulo (USP), State University Paulista campus São José dos Campos (UNESP-SJC), and Federal University of Bahia (UFBA).

- Eligibility criteria for participant selection

The inclusion criteria consisted of students enrolled in Stricto Sensu postgraduate programs in OP and OM at the aforementioned educational institutions, who voluntarily agreed to participate in the research after reading and accepting the Informed Consent Form. All master's and PhD students were included, regardless of their year of study. The exclusion criteria included students who did not fully complete the questionnaire, those not affiliated with the participating institutions, or students from other countries, even if enrolled in the participating institutions.

- Design and structure of the digital questionnaire

A digital questionnaire (Supplement 1) was created by the research group using the Google Forms platform®, containing 29 questions organized into three sections: sociodemographic profile, motivations for pursuing postgraduate studies, and satisfaction and job market prospects. To ensure the suitability of the instruments for the target audience, methodological adaptations were made based on validated scales previously used to assess feelings and behaviors in academic contexts. The formulation of the questions followed a process of review and adjustments, conducted with the collaboration of a psychologist who was part of the research group. Before the final application, a pre-test was carried out with a pilot group of postgraduate students to verify the clarity, coherence, and applicability of the items. The feedback obtained allowed final adjustments to the questionnaire, ensuring its validity and suitability for the study.

The first section explored variables including age, gender, ethnicity, marital status, the graduate's home institution, the nature of the institution, the degree being pursued (master's or PhD), the current institution and state of residence, employment status, and scholarship receipt.

The second section utilized a Likert-type scale to examine the motivations for pursuing postgraduate studies. Participants rated their motivations on a scale ranging from “0” (not important at all) to “4” (very important). These motivations included financial considerations, the desire to acquire and enhance knowledge, personal satisfaction, the influence of teachers and family, the aspiration to provide better quality service to patients, and the intention to improve one’s quality of life.

In the third section, a Likert-type scale was also used to assess participants' satisfaction levels, divided into four dimensions: chosen field of study (“1” for very dissatisfied and “5” for very satisfied), the course's pedagogical project (“1” for poor and “5” for excellent), the institution's infrastructure and physical resources (“1” for poor and “5” for excellent), and the teaching staff (“1” for poor and “5” for excellent). Additionally, their expectations regarding the job market (“1” for very negative and “5” for very positive), confidence in their preparation to enter the job market (“1” for not prepared at all and “5” for very prepared), and preferred areas of interest, such as teaching, research, and clinical practice, were analyzed. In this session, there was a question regarding satisfaction with access to the physical resources offered by the program's Institution, with the answer options being "Yes" and "No".

Data collection was conducted between August and October 2024. To reach the students, a faculty member from each institution was responsible for contacting them via email and social media platforms, such as WhatsApp, and sharing the questionnaire. These strategies were chosen due to their widespread use among postgraduate students, their efficiency in quickly reaching the target audience, and their ability to form homogeneous samples, as demonstrated by previously (11).

- Variables and statistical analysis

After application of the questionnaire, the data was tabulated and analyzed using SPSS software (Statistical Package for the Social Sciences, version 21.0, Chicago, USA) for statistical analysis. Initially, independent variables with Likert-type response options (reason for the course, expectations regarding the job market, satisfaction, confidence and preparedness for the job market, and areas of interest) were dichotomized into "No" (0 to 3) and "Yes" (4 to 5). Thus, categorical variables were described in relative and absolute frequencies.

For the inferential analyses, the Kolmogorov-Smirnov test was used to assess the normality of the data for the dependent variables (dimensions of satisfaction). The Student’s t-test was applied to compare means between variables with dichotomous response options, and ANOVA was used for more than two categories. When the variables did not follow a normal distribution, the Mann-Whitney and Kruskal-Wallis tests were the corresponding non-parametric tests employed. All analyses adopted a significance level of 5% (*p-value* < 0.05).

## Results

- Sociodemographic characteristics

The final sample consisted of 139 students who answered the questionnaire completely. The participants were predominantly female (63.3%) and mostly in the 25-30 age group (59.7%) (Table 1). In terms of educational level, the majority were studying for a PhD (55.4%), while 44.6% were studying for a master's degree. The University of Campinas (34.5%) and the state of São Paulo (58.3%) had the highest number of postgraduate students in OP and OM. Most participants declared themselves to be white (62.6%), single (77.7%) and childless (92.1%).

The majority of participants reported not working during their studies (60.4%), while 39.6% had some kind of job, predominantly private (n = 34; 24.5%), followed by public service (n = 11; 7.9%) and public/private (n = 9; 6.5%). About funding, 85.6% had a scholarship, most of which was granted by the Coordination for the Improvement of Higher Education Personnel Foundation - CAPES (78%), followed by state agencies (19.5%) and the National Council for Scientific and Technological Development - CNPq (2.4%). As for professional aspirations, 84.2% expressed interest in working in teaching in the future, while interest in research was lower (48.2%), as was interest in the clinical field (43.2%).

Geographically, the students came from different regions of Brazil, with a significant concentration in the state of São Paulo (22.3%), followed by Minas Gerais (18.7%) and Bahia (14.4%). Most of the participants graduated in the state of São Paulo (28.1%), followed by Minas Gerais (18%) and Bahia (14.3%), with less representation from states in the North, South and Midwest regions. The majority obtained their training at public higher education institutions (78.4%). Regarding the highest level of education completed, more than half (56.1%) had a master's degree, while 29.5% had an undergraduate degree and 14.4% had completed a specialization or residency.

- Motivations for a postgraduate course and job market perspectives

Regarding the reasons for pursuing postgraduate studies, 120 participants (86.3%) aimed to acquire and/or complement knowledge gained during their previous educational level, 118 (84.9%) reported personal satisfaction, and 71 (51.1%) cited financial motivations. Additionally, 100 participants (71.9%) indicated their intention to provide services to the community outside the university environment, 78 (56.1%) were influenced by professors, 31 (22.3%) by family members, and 77 participants (55.4%) mentioned quality of life as a motivating factor.

In terms of the job market, 68.3% of participants assessed that there are limited employment opportunities in their field of study. Among them, 37.4% (n = 52) of students rated their job prospects in OP/OM as good or excellent, 39.6% (n = 55) were neutral, and 23% (n = 32) rated them as poor or very poor. Furthermore, 64% of participants stated that they felt prepared to face the job market in OP/OM.

- Satisfaction with the postgraduate course

Satisfaction with the choice to pursue postgraduate studies in OP/OM was reported by 87.8% of the sample (n = 122), followed by 82% (n = 114) of participants who positively assessed access to the resources provided by the program's institution, 79.9% (n = 111) who were satisfied with the course's pedagogical project, 78.4% (n = 109) satisfied with the physical infrastructure, and 70.5% (n = 98) with access to the program's professors, both for clarifying doubts and for research collaborations.

The analysis of the associations between the dimensions of satisfaction among postgraduate students in OP/OM (Table 2) and the independent variables revealed that the level of education (master's or PhD) did not have a significant influence on students' perceptions of any of the evaluated dimensions. However, age group was associated with satisfaction with the postgraduate program (*p* = 0.020), with greater satisfaction reported by students over 40 years old. Ethnicity was associated with the pedagogical project (*p* < 0.001), with white students reporting higher levels of satisfaction. Marital status also influenced satisfaction, with partnered students demonstrating higher satisfaction with access to professors (*p* = 0.004). Additionally, students in their first year of study (2024) reported greater satisfaction with the program (*p* = 0.008) and physical infrastructure (*p* = 0.048).

Having children was also a relevant variable, associated with satisfaction with the program (*p* = 0.048) and access to professors (*p* < 0.001). Professional occupation positively influenced satisfaction with access to professors (*p* = 0.012), while students without scholarships reported higher satisfaction in this same dimension (*p* < 0.001). Employment prospects were associated with almost all evaluated dimensions, including the program (*p* < 0.001), pedagogical project (*p* < 0.001) and physical infrastructure (*p* = 0.034), indicating that good or excellent prospects are directly related to higher satisfaction levels. The perception of preparedness for the job market was also a relevant factor, positively associated with satisfaction dimensions related to the program (*p* = 0.016), pedagogical project (*p* = 0.001) and access to professors (*p* < 0.001). Moreover, the perception of job opportunities influenced satisfaction with the program (*p* = 0.015), with students who were optimistic about employment opportunities reporting higher satisfaction in this dimension.

## Discussion

This is the first multicenter study that provides an in-depth analysis of the profiles and professional satisfaction of postgraduate students in OP and OM in Brazil. The findings not only contribute to understanding the dynamics of postgraduate education in these fields but also provide valuable information for policymakers and educators to improve the structure, accessibility, and impact of these programs. Addressing labor market challenges and promoting professional integration in OP and OM remain crucial for the long-term development of these specialties.

We identified a high prevalence of satisfaction with OM/OP postgraduate programs (87.8%), which exceeds the rates reported in various other medical and dental specialties. For instance, Canadian oral and maxillofacial surgery residents reported an 80% satisfaction rate (12), Asian internal medicine residents showed an 81.44% satisfaction rate (13), and postgraduate students in orthodontics globally demonstrated only 32.55% satisfaction (14). Similarly, satisfaction among dental residents was reported to be less than 42% across all dimensions (15).

This high level of satisfaction among OM/OP postgraduate trainees aligns with findings from Villa *et al*. (2018) (16), who reported a high satisfaction score of 8 (on a 0 - 10 scale) among OM specialists. Satisfaction levels may be linked to training, a variety of career paths, combined with opportunities for assistance, teaching and research. However, the global presence and importance of OM still receives little recognition as a specialty.

It is necessary that reasons for choosing OM are identified during graduation, so that satisfaction with the specialization is consistent with the students' perspectives and expectations. Our study revealed that the main reasons for pursuing postgraduate studies were the acquisition of knowledge (86.3%), personal satisfaction (84.9%), and the desire to contribute to the community (71.9%). In contrast, in the study by Rock *et al*. (2023) (17) with 50 Canadian dental hygienists, career opportunities expansion was the most cited motivating factor (74%), followed by additional education (24%) and convenience (16%). Furthermore, 8% of participants sought postgraduate education to leave clinical practice, driven by dissatisfaction and physical wear. Despite the differences, both studies highlight the importance of acquiring knowledge, with Brazilians showing more than three times this motivation. Furthermore, the reasons for choosing a postgraduate degree are related to future work prospects.

In the national context, most graduates in OP and OM work as professors or are involved in teaching and research activities (44.3%), while 37.3% work in clinical practice (7). Despite the high interest of participants in this study in pursuing an academic career (84.2%), it is observed that the job market appears unable to absorb the growing number of professionals interested in this field. This may explain the lower proportion of aspirations focused on research (48.2%) and clinical practice (43.2%), which emerge as alternatives considering the limitations of the academic job market.

About the profile of OP/OM postgraduate students, there was a higher participation of female students (63.3%). This finding aligns with a previous study conducted by the Brazilian Society of Oral Medicine and Pathology (SOBEP), which evaluated the participation of female researchers in OP/OM. It was observed that, among the 197 professionals with active registration in 2023, 117 (59.4%) were women (18). Thus, although no significant difference was found between satisfaction and gender among students, the substantial female participation in both specialties in the country stands out.

The Southeast region of Brazil concentrates many postgraduate students and researchers, especially in the state of São Paulo, which hosts 58.3% of postgraduates (19,20). The University of Campinas stands out as the leading institution, with a 34.5% share, being the only public institution with a Stricto sensu postgraduate program exclusively in PO and MO, establishing itself as a reference center. Furthermore, the Southeast region is home to 44.28% of the 70 higher education institutions that offer postgraduate programs in Dentistry, with most master's and PhD programs, as well as the highest-rated ones, located in this region (https://sucupira.capes.gov.br/sucupira/). The historical centralization of financial resources, infrastructure, and more robust funding policies, such as those from the noTable São Paulo Research Foundation (FAPESP), along with the availability of high-quality programs, explains the predominance of this region both in terms of the number and quality of academic and scientific training in the field of Dentistry (6).

We observed that white ethnic students reported higher satisfaction compared to black students. This disparity is corroborated by studies in the literature that point to possible influences of racial bias in the academic environment. A meta-analysis (21) found that non-white medical students performed worse academically than their white peers, suggesting that social factors may contribute to these differences. Additionally, Klein *et al*. (2022) (22) found that racial minority residents received significantly lower clinical evaluations, which may reflect implicit biases on the part of professors and advisors. In this same scenario, a study with medical residents found that 45% of students witnessed episodes of harassment and racism during their training, indicating an environment that can negatively affect the satisfaction of black students (23). These findings suggest that structural racism and relationships with teachers can directly influence student satisfaction, especially regarding teacher access and support, the dimension that presented the lowest average value in our analysis.

Supervision plays an important role in improving the learning environment and is often cited as one of the main factors influencing both the successful completion of graduate programs and students’ academic experience (24). In this study, married students (*p* = 0.004), those with children (*p* < 0.001), those who worked (*p* = 0.012), those who did not receive scholarships (*p* < 0.001), and those who felt prepared for the job market (*p* < 0.001) reported greater satisfaction with their professors. These results may reflect the intrinsic characteristics of these groups, such as greater resilience, appreciation of educational opportunities, and recognition of faculty's contribution to their development. According to Beaudin *et al*. (2015) (24), a good supervisor should be accessible, maintain frequent interactions with students and offer emotional and motivational support. Furthermore, it is important to appropriately organize the workload to avoid overload and periods of inactivity. Good communication, continuous monitoring of students' progress, especially during the writing of their dissertation or thesis and guidance on career planning are essential aspects of effective supervision.

The postgraduate system in Brazil plays a crucial role in generating scientific knowledge, with most research being conducted at public universities funded by the government (25). Postgraduate programs are the main contributors to the country's scientific output, accounting for approximately 85% of it, according to CAPES (https://www.gov.br/capes/pt-br). Data from the present study indicated that 85.6% of students received scholarships, predominantly funded by CAPES (78%), followed by state agencies (19.5%) and CNPq (2.4%). However, despite this significant financial investment, no correlation was found between receiving scholarships and student satisfaction, highlighting other factors that may influence the academic experience.

These findings underscore the relevance of CAPES, linked to the Ministry of Education, as the primary agency responsible for research funding and scholarship payments in Brazil (https://www.gov.br/capes/pt-br). Nevertheless, scholarship funding and their assigned values are directly tied to the political context. In certain periods, budget cuts have impacted federal scholarships, postdoctoral programs, international collaborations, and student aid, causing instability and dissatisfaction among postgraduate students (25). This emphasizes the need for public policies that ensure the sustainability of Brazil's postgraduate system.

A previous study (26) investigating the perceptions of Dentistry students at a Brazilian state university found that 44.1% of participants considered the job market unfavorable to the profession. This negative outlook was attributed to factors such as market saturation, high competitiveness, devaluation of the field, and unfair competition practices. In this context, our study revealed that the majority (68.3%) of postgraduate students perceived limited employment opportunities. However, more than one-third (37.4%) rated their job prospects as good or excellent. This optimism was positively associated with higher satisfaction levels in key dimensions, including the academic program (*p* < 0.001) and the pedagogical project (*p* < 0.001), underscoring the importance of aligning academic experiences with professional aspirations.

The job market clearly reflects the rapid transformations occurring in the field of dentistry (27). These impacts are evident in our study, where students who considered themselves prepared for the job market reported higher satisfaction with the course (*p* = 0.016), the pedagogical project (*p* = 0.001) and access to professors (*p* < 0.001) compared to those who did not feel prepared. Similarly, participants with a positive perception of employment opportunities demonstrated greater satisfaction with the postgraduate program (*p* = 0.015). However, it is concerning to note that 34% of the participants reported not feeling prepared for the job market. This feeling of insecurity may be related to dissatisfaction with the course, potentially negatively influencing the academic trajectory, hindering the learning processes, and affecting professional development in the future (28).

The study has some limitations. The sample, although representative of various Brazilian institutions, may not fully reflect the experiences of students from underrepresented regions and private institutions. Moreover, the cross-sectional nature of the study did not allow for an assessment of changes in satisfaction perceptions over time. Despite these limitations, the findings provide a relevant and novel overview of the profile and satisfaction of graduate students in OP and OM in Brazil, offering valuable insights for improving training programs.

This multicenter study showed that students are motivated by personal satisfaction, knowledge expansion, and aspirations for teaching and research. Although they are generally positive about institutional resources, pedagogical projects, and faculty accessibility, concerns about limited job opportunities in OP and OM remain. This emphasizes the need for public policies aimed at creating new jobs in these areas of work. A strong interest in academic careers was also revealed, with teaching and research prioritized over clinical practice. Satisfaction varied based on age, ethnicity, and marital status, highlighting the need for more inclusive support. Additionally, the regional concentration of participants suggests a need for a more equiTable distribution of resources and opportunities across Brazil.

## Figures and Tables

**Table 1:** Distribution of sociodemographic and academic variables of Brazilian postgraduate students in Oral Pathology and Oral Medicine (n = 139).

Variable		N	%
Age (years)	Under 25	14	10.1
	25 to 30	83	59.7
	31 to 35	28	20.1
	36 to 40	5	3.6
	Above 40	9	6.5
Ethnicity	White	87	62.6
	Black	8	5.8
	Brown	42	30.2
	Yellow	2	1.4
	Indigenous	0	0
Marital status	Single	108	77.7
	Married	28	20.1
	Divorced	3	2.2
	Widowed	0	0
State of study	Bahia	18	12.9
	Minas Gerais	15	10.8
	Paraíba	7	5
	Rio Grande do Norte	18	13
	São Paulo	81	58.3
Current educational institution	University of Campinas	48	34.5
	Federal University of Bahia	18	12.9
	State University Paulista campus São José dos Campos	18	12.9
	Federal University of Rio Grande do Norte	18	12.9
	Federal University of Minas Gerais	15	10.8
	University of São Paulo	15	10.8
	Federal University of Paraíba	7	5.0
Year of start of the current level of education	2024	64	46
	2023	38	27.3
	2022	16	11.5
	2021	13	9.4
	2020	7	5
	2019	1	0.7
Highest level of education completed	Graduation	41	29.5
	Specialization and/or residency	20	14.4
	Master's degree	78	56.1
Interest in future work	Teaching	117	84.2
	Research	67	48.2
	Clinical field	60	43.2

**Table 2:** Association between satisfaction and independent variables of the study of postgraduate students in Oral Medicine/Oral Pathology.

Variables		Satisfaction dimensions							
		Postgraduate course		Pedagogical project		Physical structure		Access to teachers	
		Mean (SD)	p-value	Mean (SD)	p-value	Mean (SD)	p-value	Mean (SD)	p-value
Education level	Master's degree	4.29 (0.77)	0.601	4.13 (0.89)	0.652	4.13 (0.89)	0.669	3.76 (0.88)	0.350
	PhD degree	4.36 (0.87)		4.19 (0.79)		4.19 (0.90)		3.90 (0.83)	
Age group	Under 25 years old	4.57 (0.51)	0.020^?^	4.07 (0.82)	0.208	4.21 (0.89)	0.188	3.79 (0.57)	0.099
	25 to 30 years old	4.28 (0.91)		4.13 (0.90)		4.25 (0.89)		3.72 (0.84)	
	31 to 35 years old	4.18 (0.72)		4.07 (0.71)		3.82 (0.90)		3.93 (1.01)	
	36 to 40 years old	4.20 (0.83)		4.60 (0.54)		4.40 (0.89)		4.40 (0.89)	
	Over 40 years old	5.00 (0.00)		4.67 (0.50)		4.17 (0.89)		3.83 (0.85)	
Sex	Male	4.31 (0.94)	0.676	4.18 (0.76)	0.832	4.24 (0.95)	0.894	3.88 (0.76)	0.310
	Female	4.3 (0.75)		4.16 (0.88)		4.13 (0.86)		3.81 (0.90)	
Ethnicity	White	4.32 (0.88)	0.135	4.26 (0.75)	<0.001^‡^	4.25 (0.90)	0.058	3.89 (0.89)	0.333
	Black	3.75 (0.88)		3.00 (1.06)		3.38 (0.74)		3.50 (1.06)	
	Brown	4.48 (0.63)		4.17 (0.82)		4.12 (0.86)		3.83 (0.73)	
	Yellow	4.00 (1.41)		4.50 (0.70)		4.50 (0.70)		3.00 (1.41)	
Marital status	No partner	4.34 (0.84)	0.733	4.13 (0.85)	0.248	4.23 (0.84)	0.175	3.73 (0.84)	0.004*
	With partner	4.29 (0.76)		4.32 (0.77)		3.93 (1.05)		4.25 (0.79)	
Year of start of the current level of education	2019 to 2023	4.16 (0.95)	0.008*	4.07 (0.85)	0.132	4.03 (1.00)	0.048*	3.84 (0.93)	0.934
	2024	4.53 (0.59)		4.28 (0.80)		4.33 (0.73)		3.83 (0.76)	
Children	Yes	4.73 (0.64)	0.048¶	4.45 (0.68)	0.178	3.91 (1.22)	0.325	4.64 (0.50)	<0.001*
	No	4.30 (0.83)		4.14 (0.84)		4.19 (0.86)		3.77 (0.84)	
Job	Yes	4.42 (0.68)	0.289	4.29 (0.71)	0.135	4.04 (0.92)	0.176	4.05 (0.78)	0.012*
	No	4.27 (0.91)		4.08 (0.90)		4.25 (0.87)		3.69 (0.87)	
Scholarship	Yes	4.35 (0.82)	0.454	4.16 (0.86)	0.819	4.18 (0.85)	0.536	3.71 (0.80)	<0.001*
	No	4.20 (0.83)		4.20 (0.69)		4.05 (1.14)		4.55 (0.82)	
Grant agency	CAPES	4.22 (0.98)	0.769	4.08 (0.99)	0.601	4.08 (0.93)	0.194	3.69 (0.92)	0.427
	CNPq	4.50 (0.70)		4.00 (0.00)		5.00 (0.00)		4.50 (0.70)	
	State foundation	4.38 (0.50)		3.94 (0.68)		4.38 (0.71)		3.75 (0.85)	
Job prospects	Good/excelente	4.62 (0.66)	<0.001*	4.52 (0.61)	<0.001*	4.37 (0.79)	0.034*	3.96 (0.76)	0.177
	Neutral /terrible/bad	4.16 (0.87)		3.95 (0.88)		4.05 (0.93)		3.76 (0.90)	
Prepared for the job market	Yes	4.47 (0.67)	0.016*	4.35 (0.72)	0.001*	4.13 (0.90)	0.591	4.04 (0.68)	<0.001*
	No	4.08 (1.00)		3.84 (0.93)		4.22 (0.88)		3.46 (0.99)	
Perception of employment opportunities	Yes	4.57 (0.72)	0.015*	4.30 (0.73)	0.186	4.18 (0.81)	0.878	3.82 (0.81)	0.876
	No	4.22 (0.85)		4.11 (0.88)		4.16 (0.93)		3.84 (0.87)	

Associations: *t Student test. ¶Mann-Whitney test. †Kuskal-Wallis test. ‡ANOVA test.
